# Acceleration of leukocytes’ epigenetic age as an early tumor and sex-specific marker of breast and colorectal cancer

**DOI:** 10.18632/oncotarget.15573

**Published:** 2017-02-21

**Authors:** Danielle Fernandes Durso, Maria Giulia Bacalini, Claudia Sala, Chiara Pirazzini, Elena Marasco, Massimiliano Bonafé, Ítalo Faria do Valle, Davide Gentilini, Gastone Castellani, Ana Maria Caetano Faria, Claudio Franceschi, Paolo Garagnani, Christine Nardini

**Affiliations:** ^1^ Department of Experimental, Diagnostic and Specialty Medicine, Alma Mater Studiorum-University of Bologna, Bologna, Italy; ^2^ National Counsel of Technological and Scientific Development (CNPq), Ministry of Science Technology and Innovation (MCTI), Brasilia, Brazil; ^3^ IRCCS Istituto delle Scienze Neurologiche di Bologna, Bologna, Italy; ^4^ Department of Physics and Astronomy, University of Bologna, Bologna, Italy; ^5^ Istituto Auxologico Italiano IRCCS, Cusano Milanino, Milan, Italy; ^6^ Biochemistry and Immunology Department, Biological Sciences Institute, Federal University of Minas Gerais, Belo Horizonte, Brazil; ^7^ Applied Biomedical Research Center, S. Orsola-Malpighi Polyclinic, Bologna, Italy; ^8^ Interdepartmental Center “L. Galvani”, University of Bologna, Bologna, Italy; ^9^ Clinical Chemistry, Department of Laboratory Medicine, Karolinska Institutet at Huddinge University Hospital, Stockholm, Sweden; ^10^ Personal Genomics S.r.l., Verona, Italy; ^11^ CNR IAC “Mauro Picone”, Rome, Italy

**Keywords:** epigenetic clock, ELOVL2, FHL2, cancer, blood

## Abstract

Changes in blood epigenetic age have been associated with several pathological conditions and have recently been described to anticipate cancer development. In this work, we analyze a publicly available leukocytes methylation dataset to evaluate the relation between DNA methylation age and the prospective development of specific types of cancer. We calculated DNA methylation age acceleration using five state-of-the-art estimators (three multi-site: Horvath, Hannum, Weidner; and two CpG specific: ELOV2 and FHL2) in a cohort including 845 subjects from the EPIC-Italy project and we compared 424 samples that remained cancer-free over the approximately ten years of follow-up with 235 and 166 subjects who developed breast and colorectal cancer, respectively. We show that the epigenetic age estimated from blood DNA methylation data is statistically significantly associated to future breast and male colorectal cancer development. These results are corroborated by survival analysis that shows significant association between age acceleration and cancer incidence suggesting that the chance of developing age-related diseases may be predicted by circulating epigenetic markers, with a dependence upon tumor type, sex and age estimator. These are encouraging results towards the non-invasive and perspective usage of epigenetic biomarkers.

## INTRODUCTION

Cancer is an age related disease [1–5]. Consequently, exploration of the association between markers of ageing and cancer represents an obvious step to bring advances in both research areas.

Biomarkers that linearly change with chronological age are now available to the scientific community and span from anatomical (e.g. ocular biomarkers [6]) to molecular ones including micro-RNAs levels [7, 8], protein modifications [9] and telomeres’ length [10].

DNA methylation-based biomarkers have gained relevance in the last few years for many reasons. First, both genome-wide and high-throughput targeted approaches to measure DNA methylation are easily accessible and highly reproducible. Second, these markers show extremely high correlation with chronological age and with age-acceleration effects associated with pathological conditions, morbidity and mortality. Taken together, these results make it possible to hypothesize that a positive deviation from normal aging trajectories (i.e. higher biological than chronological age) could be predictive, if not causative, of the development of several diseases, including cancer [11].

To date a few studies have approached this idea, with still inconclusive results. Nan et al. found no association between the overall white blood cell (WBC) DNA methylation levels and colorectal cancer (CRC) risk among 358 females where blood samples had been collected prior to CRC diagnosis [12]. On the contrary, Pufulete et al. [13] and Lim et al. [14] reported significant association between hypomethylation in WBC DNA and an increased risk for colorectal adenomas. Finally, Walters et al. [15] described correlation between three DNA repetitive elements that present increased methylation levels in WBC from 539 cases diagnosed before 60 years of age and 242 healthy, cancer free, subjects.

Because of their ease of calculation and their prognostic potential, several methodologies have been developed to compute the epigenetic age.

Horvath's epigenetic clock [16, 17], a multi-tissue predictor based on the methylation status of 353 CpG sites assessed by the Infinium HumanMethylation27 BeadChip (HM27) is among the most popular epigenetic age estimators. According to Horvath's clock, age-acceleration was found in blood, brain and saliva from people affected by Down syndrome, a disease characterized by atypical aging patterns [18]. Similarly, the same clock successfully detected age acceleration in dorsolateral prefrontal cortex from patients with Alzheimer's disease [19] and in whole blood from Parkinson's disease patients [20]. Frailty [21], lifetime stress [22], HIV-1 infection [23], and menopause [24] were also found to accelerate epigenetic age of WBC. Finally it was demonstrated that epigenetic age estimated from whole blood DNA methylation is correlated to physical and cognitive fitness [25] and mortality [26–30] in large human cohorts. Importantly, Horvath's clock is able to detect not only age-acceleration, but also age-deceleration effects in models of healthy aging and longevity [31].

Another epigenetic age-associated biomarker has been developed by Hannum et al. [29] and relies on the DNA methylation values of 71 CpG sites (only six being in common with Horvath's) from the Infinium HumanMethylation450 BeadChip (HM450). Differently from Horvath's clock, Hannum's model was calibrated on whole blood only. Three studies demonstrated the association of this epigenetic clock with biological fitness and mortality [25, 26, 28], as well as its association with post-traumatic stress disorders [32].

The quantification of age acceleration (and deceleration) starting from easy-to-access blood samples has triggered efforts towards the simplification of Horvath's and Hannum's epigenetic clocks.

In this direction, a model based on 3 CpG sites (Weidner's estimator) was found to significantly correlate with chronological age [33] but failed to predict mortality in the Lothian Birth Cohort 1921 study [34].

Finally, our group identified two HM450 CpG probes, cg16867657 in the CpG island of *ELOVL2* and cg06639320 in the CpG island of *FHL2* showing very high correlation with chronological age (Spearman correlation = 0.91) in whole blood DNA methylation data [35, 36]. These two loci were confirmed in several replicative tissues other than blood [36–40] and have been calibrated so far on teeth samples [41].

Age acceleration phenomena have been investigated also in cancer patients, owing to the peculiar observation that biomarkers of aging do not systematically show age acceleration in the tumour tissues, while they do in the blood of cancer-free people who develop cancer prospectively [42, 43]. To confirm an expand these promising findings we explored the reproducibility of this observation in an independent cohort collected by the Human Genetics Foundation (HuGeF, Turin, Italy) including prospective breast cancer and CRC data [44].

## RESULTS AND DISCUSSION

Epigenetic age was estimated from DNA methylation blood data using 5 different methodologies: Horvath's, Hannum's, Weidner's, *ELOVL2* and *FHL2* DNA methylation ages (DNAmAges) with and without adjustment for blood cell counts. We will use the term “Age Accel” to refer to non-adjusted age acceleration and IEAA otherwise. See Material and Methods for details.

**Figure 1:**
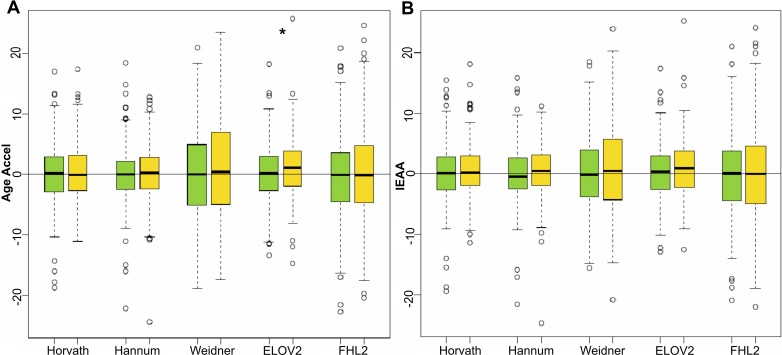
Age acceleration predictors in breast cancer samples Boxplots of Age Accel (**A**) and IEAA (**B**) values for 233 female control subjects (green) and 233 female subjects that developed breast cancer at follow up (yellow), estimated by the 5 epigenetic predictors. Asterisks indicate significant differences according to Mann-Whitney-Wilcoxon test (*p*-value < 0.05), which was 0.0432 for ELOVL2 age acceleration estimators.

Age Accel between females that developed breast cancer at follow-up and controls (cancer-free patients) was statistically significantly different only when using the *ELOVL2* clock (Mann-Whitney-Wilcoxon test *p*-value = 0. 0432), with Age Accel values in tumor samples on average 0.9 years higher than in the control group (Supplementary Table 1 and Figure 1). Despite conservation of this trend (i.e. subjects that developed breast cancer still tend to have higher *ELOVL2*-based IEAA values than controls) statistical significance was lost when correction for blood cell counts was applied.

**Figure 2:**
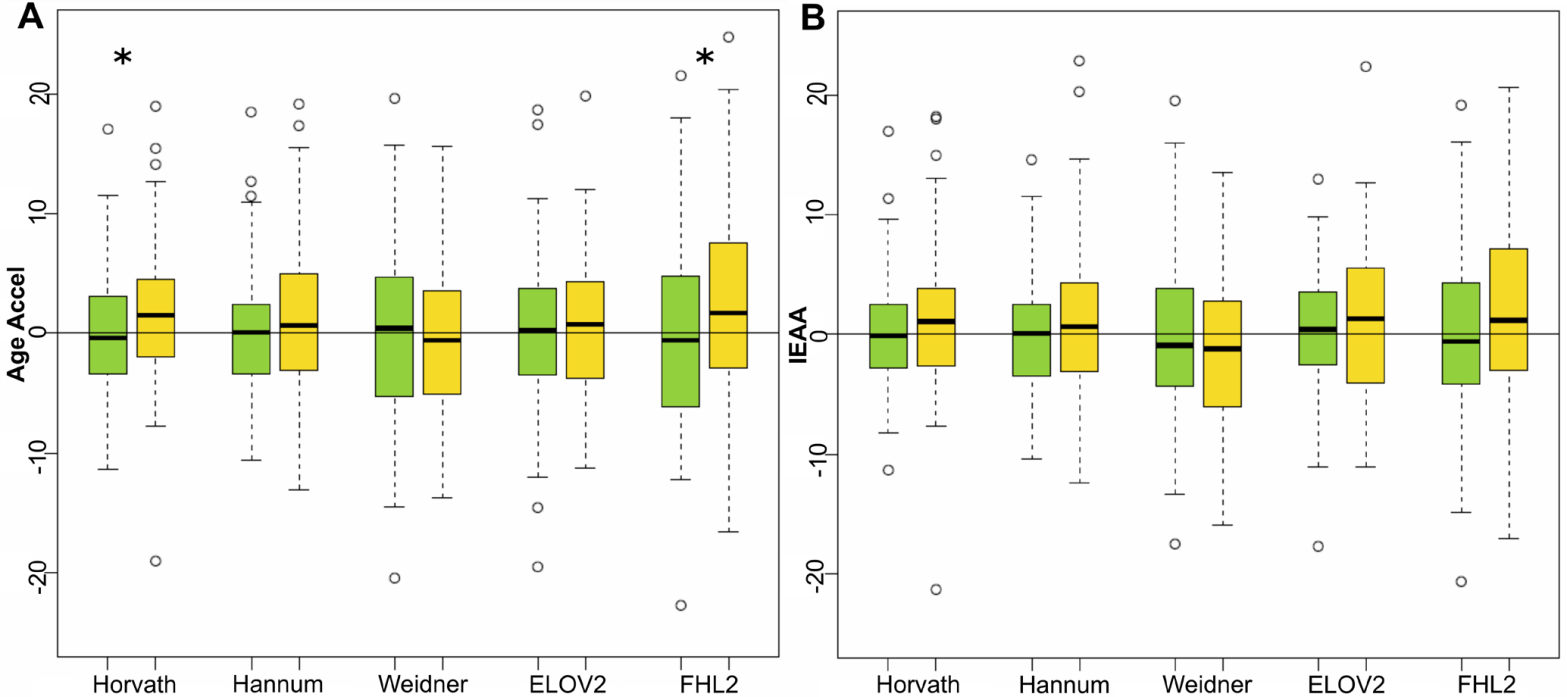
Age acceleration predictors in colorectal cancer male samples Boxplots of Age Accel (**A**) and IEAA (**B**) values for 84 male control subjects (green) and 87 subjects that developed CRC at follow up (yellow), estimated by the 5 epigenetic predictors. Asterisks indicate significant differences according to Mann-Whitney-Wilcoxon test (*p*-value < 0.05), which were respectively 0.0421 and 0.0363 for Horvath and FHL2 age acceleration estimators.

With respect to the male subjects that developed CRC, Horvath's and *FHL2* clocks returned a significant increase in Age Accel values (Mann-Whitney-Wilcoxon test *p-value* = 0.0421 and 0.0363 for Horvath's and *FHL2*'s estimations respectively). Subjects that developed colon cancer were 1.6 and 2.5 years older using Horvath and *FHL2* methods than their respective controls (Supplementary Table 1 and Figure 2). Although results by Hannum's clock showed an evident trend towards higher Age Accel, this predictor did not give significant results, nor did Weidner's nor *ELOVL2* clocks. None of the 5 methods returned significant differences when IEAA values were compared, although a trend was visible with Horvath's, Hannum's and *FHL2* clocks.

**Figure 3:**
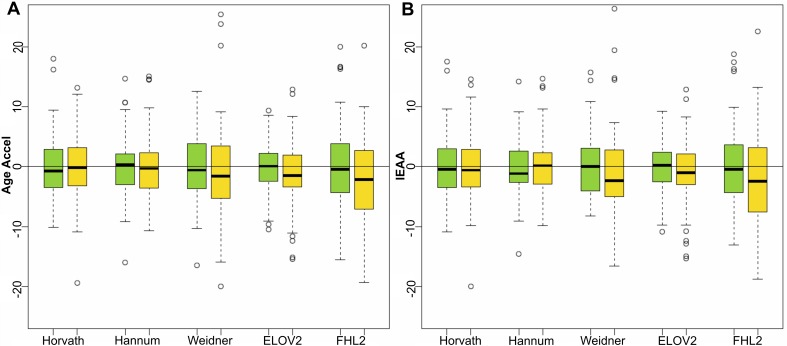
Age acceleration predictors in colorectal cancer female samples Boxplots of Age Accel (**A**) and IEAA (**B**) values for 79 female control subjects (green) and 79 subjects that developed breast cancer at follow up (yellow), estimated by the 5 epigenetic predictors.

For the CRC female counterpart, no significant differences were observed for Age Accel nor IEAA in any of the 5 predictors, despite a visible difference between the medians for Weidner, *FHL2* and *ELOV2* estimators (Supplementary Table 1 and Figure 3).

**Figure 4:**
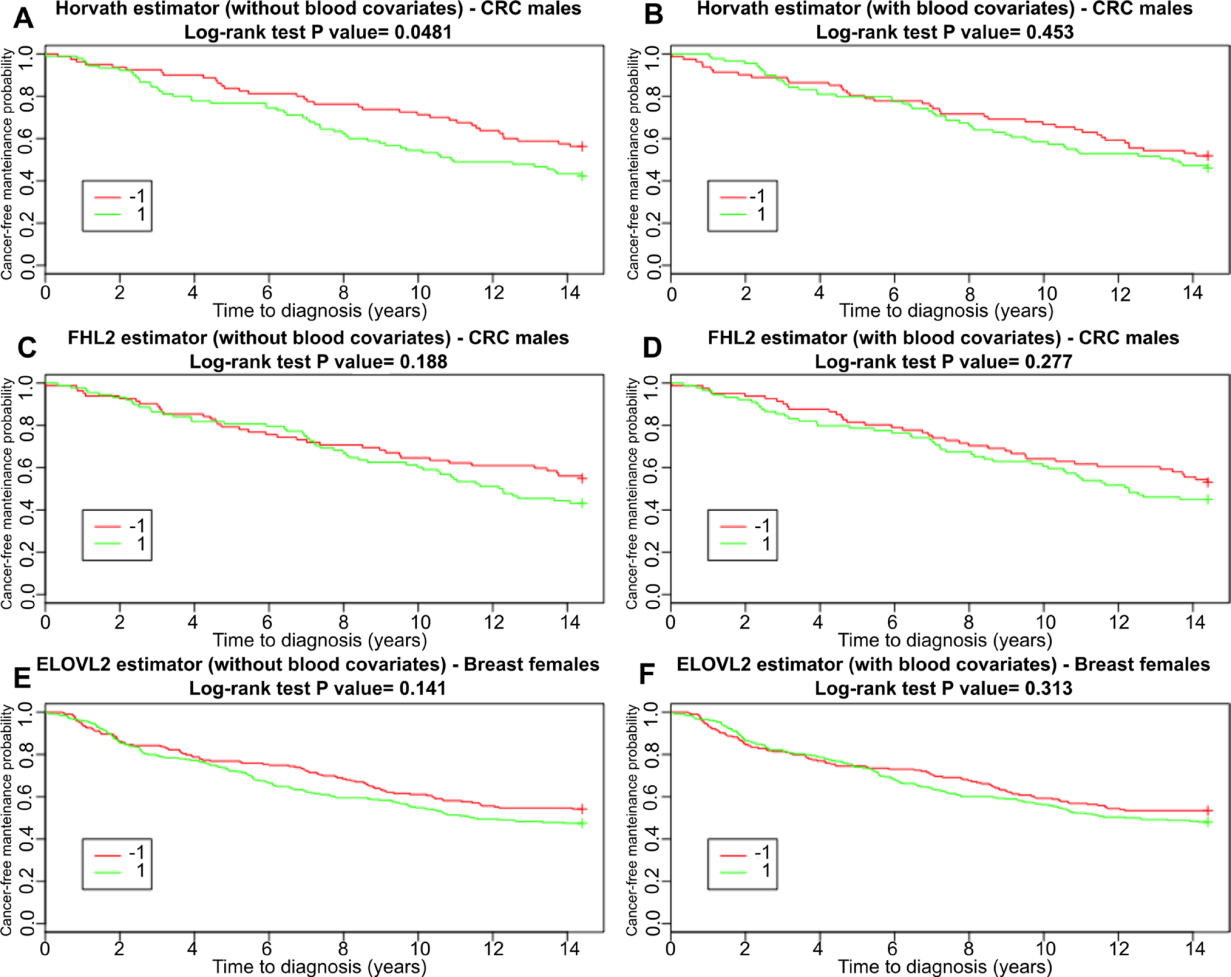
Survival functions for subjects belonging to the CRC males and breast cancer groups (including controls) incidence estimated with Kaplan-Meier method Results are shown separately for accelerated (1) and decelerated (−1) age subjects, with age acceleration computed considering the estimators that showed significant differences between cases and controls: Horvath and FHL2 estimator for the CRC males dataset (**A**–**D** charts) and ELOVL2 for the breast dataset (**E**–**F** charts). In each chart title, we reported the Log-Rank test *p*-values comparing survival curves.

To explore these results further, we performed survival analysis using Kaplan-Meier method. For each of the five DNAmAge estimators we considered both Age Accel and IEAA values. Figure 4 shows the results for Age Accel and the corresponding IEAA obtained with Horvath, FHL2 and ELOV2, which are the estimators that were able to reveal significant differences in age acceleration between tumor and control samples. Results relative to all the other clocks and subgroups are reported in Supplementary Figures 1, 2 and 3. Log-rank test *p*-values are summarized in Table 1.

**Table 1:** Survival analysis

	Horvath		Hannum		Weidner		ELOVL2		FHL2	
	Age Acc	IEAA	Age Acc	IEAA	Age Acc	IEAA	Age Acc	IEAA	Age Acc	IEAA
BRC females	0.581	0.786	0.429	0.216	0.206	0.212	0.141	0.313	0.841	0.885
CRC males	0.0481	0.453	0.346	0.68	0.313	0.527	0.767	0.231	0.188	0.277
CRC females	0.732	0.67	0.541	0.202	0.479	0.165	0.0424	0.0395	0.423	0.479

Log-Rank test *p*-values for the three studied datasets and considering all five epigenetic age estimators, with and without correction for blood cell counts.

Overall, our analysis expands the results of Levine et al. focusing on lung cancer development using only Horvath's epigenetic clock [43], and of Zheng et al. who applied both Horvath's and Hannum's predictors to a cohort of subjects that prospectively developed different types of cancer (mainly skin and prostate cancer) [42] and found that blood epigenetic age is related to cancer development and could be a potential biomarker for cancer early detection.

Here we observed that the two most used epigenetic clocks, Horvath's and Hannum's, are unable to detect age acceleration effects in blood of females that were later diagnosed with breast cancer, while significant differences were observed with *ELOVL2* predictor. On the contrary, age acceleration computed with Horvath's epigenetic clock, together with *FHL2* clock, were associated with CRC development in males [42, 43].

The biological reasons behind the effectiveness of each clock is still to be unveiled, although the diverse epigenetic origin of each tumor type is bound to impact on the definition of CpG specific age acceleration.

In conclusion, we showed that different epigenetic estimators identify age acceleration effects in whole blood of subjects that prospectively developed cancer with a tumor type- and sex-specificity. These results reinforce the idea that a surrogate tissue can be used to evaluate the susceptibility to develop age-related diseases in other tissues and are encouraging for the fine tuning of more precise prognostic epigenetic biomarkers of age. In this sense, the observation that single CpG predictors, like ELOVL2 and FHL2, can detect epigenetic age deviations associated with future diagnosis of specific cancer types is of practical relevance.

On the cautious side, it is known that several variables like behavioral habits or previous health information (recently reviewed in [45]) may act as confounders of epigenetic age estimative. Therefore, the limited number of such variables made available (including batches) could be a limitation of this work. Given the potential of such results, a higher number of prospective studies of this type with freely accessible data is crucially needed to independently validate these findings.

## MATERIAL AND METHODS

### Blood dataset

We interrogated the Gene Expression Omnibus (GEO) repository using the search terms *GPL13534* (GEO identifier for the HM450 platform)*, cancer* and *follow up*. On February 2017, this search output 5 datasets, among those we selected the only one (GSE51032) whose sample size (hundreds of patients) is able to guarantee robustness of all findings and sufficient statistical power. The GSE51032 dataset contains DNA methylation measures on blood cells (buffy coats) from subjects that were prospectively followed by the Human Genetics Foundation (HuGeF) in Turin, Italy as part of the European Prospective Investigation into Cancer and Nutrition (EPIC). This study was conducted in ten European countries on populations that differ markedly in terms of dietary habits and cancer risk. The Italian EPIC cohort consists of 47,749 people recruited in the centers of Ragusa (6,404 subjects), Florence (13,597 subjects), Turin (10,604 subjects), Naples (5,062 subjects, women only) and Milan (12,079 subjects) [46, 47]. In Turin, the study recruitment began in 1993–1998 (people were aged 35-64 with no previous cancer) and was closed in 2010. The dataset includes DNA methylation data from 845 participants, selected as follows: 188 men and 657 women; at final follow-up 424 remained cancer-free (*control samples*), 235 had developed primary breast cancer, 166 had developed CRC and 20 had developed other primary cancers (5 bladder, 4 prostate gland, 4 skin, 2 bronchus and lung, 1 hemato reticuloendothelial, 1 corpus uteri, 1 kidney, 1 thyroid and endocrine glands, 1 unknown primary site lesion). In this work, we grouped colon, rectosigmoid and rectum data under the unifying label of CRC. To guarantee reproducibility and statistical power of our analyses, epigenetic age was calculated only for breast and CRC (>150 samples each).

All samples characteristics are reported in Table 2. Females and males were analyzed separately (stratification approach), for the two types of tumors, according to the recent report on sex-related differences in epigenetic age predictions [48]. Sex, in fact, is a confounding variable that affects both tumor incidence and age acceleration, and stratification has the advantage to take this into account, as well as to estimate the association between age acceleration and tumor incidence separately for females and males. Finally, to avoid unequal sample size issues, we randomly selected a subgroup of control samples with the same size and the same mean age of the group under study (Table 2).

**Table 2:** Sample characteristics

	*N*	Age at recruitment (mean years ± sd)/Median	Time to diagnosis (mean years ± sd)/Median	Wilcox test on Age at recruitment (*p*-value)
All female control samples	340	52.57 ± 7.4/(53.30)	-	
All male control samples	84	55.89 ± 5.6/(56.72)	-	
Selected breast female controls	233	52.57 ± 7.4/(53.27)	-	0.8678
Breast female cases	233	52.37 ± 7.4/(53.70)	3.84 ± 2.87/(2.69)	
CRC male controls	84	55.89 ± 5.6/(56.72)	-	0.8821
CRC male cases	87	55.97 ± 5.7/(56.53)	-	
Selected CRC female controls	79	53.71 ± 6.9/(53.71)	-	0.7306
CRC female cases	79	54.09 ± 7.6/(54.25)	5.11 ± 2.59/(4.99)	

Descriptive characteristics of the study samples. Mann-Whitney-Wilcoxon test was performed between each pair of cse and control samples to show that there were not differences between their chronological ages.

As reported more in details at https://www.ncbi.nlm.nih.gov/geo/query/acc.cgi?acc=GSE51032, genomic DNA was extracted and purified from peripheral blood leukocytes and bisulfite converted before being amplified, fragmented and hybridized to Illumina Infinium HumanMethylation450 BeadChips finally imaged using standard protocols and settings.

### Estimation of DNAmAge

We used five methodologies to estimate the epigenetic age (DNA methylation age, DNAmAge) in blood samples.

Horvath and Hannum DNAmAge were calculated using the online tool available at https://dnamage.genetics.ucla.edu/ [16]. The tool also provides counts estimates of naive CD8 T cells, exhausted CD8 T cells, plasma B cells (effector B cells), CD4 T cells, natural killer cells, monocytes, and granulocytes [43]. These estimates can be used to correct the DNAmAge taking into account possible variations due to the heterogeneity in blood cell counts between individuals (i.e. estimated cells abundance acting as covariates [31]). As mentioned above, we denote the non-adjusted epigenetic age acceleration as Age Accel, and we use the term IEAA (Intrinsic Epigenetic Age Acceleration of blood) when referring to regression residuals corrected by blood cell counts, in accordance with Horvath's nomenclature [20].

Weidner's epigenetic clock: Weidner et al. [33] generated a multivariate model based on the methylation values at 3 HM450 probes (α: cg02228185; β: cg25809905; γ: cg17861230). Weidner's DNAmAge was calculated using the equation DNAmAge = 38.0–26.4 * α 23.7 * β + 164.7 * γ [33].

*ELOVL2* and *FHL2* [35]: linear regressions between beta values of each of the two probes and chronological age were computed on the Hannum's dataset, resulting in the following models: *ELOVL2* DNAmAge = 158.81 * (cg16867657 beta value) – 42.35; and *FHL2* DNAm Age = 198.6 * (cg06639320 beta value) -30.12.

For Weidner, ELOVL2 and FHL2 clocks, we considered blood cell count adjusted and non-adjusted age acceleration (IEAA and Age Accel respectively), using the same cell counts estimates returned by Horvath's online tool.

### Statistical analysis

For each of the above-mentioned age predictors, we used regression analysis to calculate the relation between chronological age and DNAmAge in the control group. We fitted the model without including the tumor samples to obtain positive age acceleration for subjects whose epigenetic age is higher than the control group, chronological age being equal.

For each sample, the regression residuals provide an estimate of the epigenetic age acceleration (Age Accel and IEAA) in relation to the control group [16, 31].

Differences in age acceleration between controls and subjects who developed tumors were tested using the Wilcoxon-Mann-Whitney method to comply with the imperfect adherence to normality of the data.

The association between age acceleration and cancer incidence was evaluated through survival analysis, and performed considering all five epigenetic clocks. Survival functions for the accelerated (1) and decelerated (−1) age groups were fitted with Kaplan-Meier method.

Since the dataset does not provide the exact enrollment time for the control subjects, but specifies that they were recruited between 1993 and 1998 and that they were all followed up until 2010, we considered for the controls (censored data) a survival time of 14.5 years, that corresponds to an average recruitment time.

All statistical analyses and graphics were produced using the computing environment R.

## SUPPLEMENTARY MATERIALS TABLE AND FIGURES


