# Supplementary material for: Acceleration of leukocytes’ epigenetic age as an early tumor and sex-specific marker of breast and colorectal cancer
Source: Oncotarget. 2017 Feb 21;8(14):23237–45. doi: 10.18632/oncotarget.15573 (PMC5410300; doi:10.18632/oncotarget.15573)
Supplement: Supplementary file 1 [file oncotarget-08-23237-s001.pdf]

Acceleration of leukocytes' epigenetic age as an early tumor- and sex-specific marker of breast and colorectal cancer

Supplementary Materials

Supplementary Table 1: Regression *p* values

	Horvath		Hannum		Weidner		ELOVL2		FHL2	
	Age Acc	IEAA	Age Acc	IEAA	Age Acc	IEAA	Age Acc	IEAA	Age Acc	IEAA
BRC females	0.635	0.433	0.426	0.135	0.242	0.266	0.043	0.065	0.089	0.941
CRC males	0.421	0.164	0.128	0.369	0.712	0.283	0.571	0.322	0.036	0.097
CRC females	0.840	0.958	0.797	0.805	0.380	0.186	0.152	0.223	0.139	0.117

P values from regression models for each epigenetic clock estimator (with and without blood cells counts as covariates).

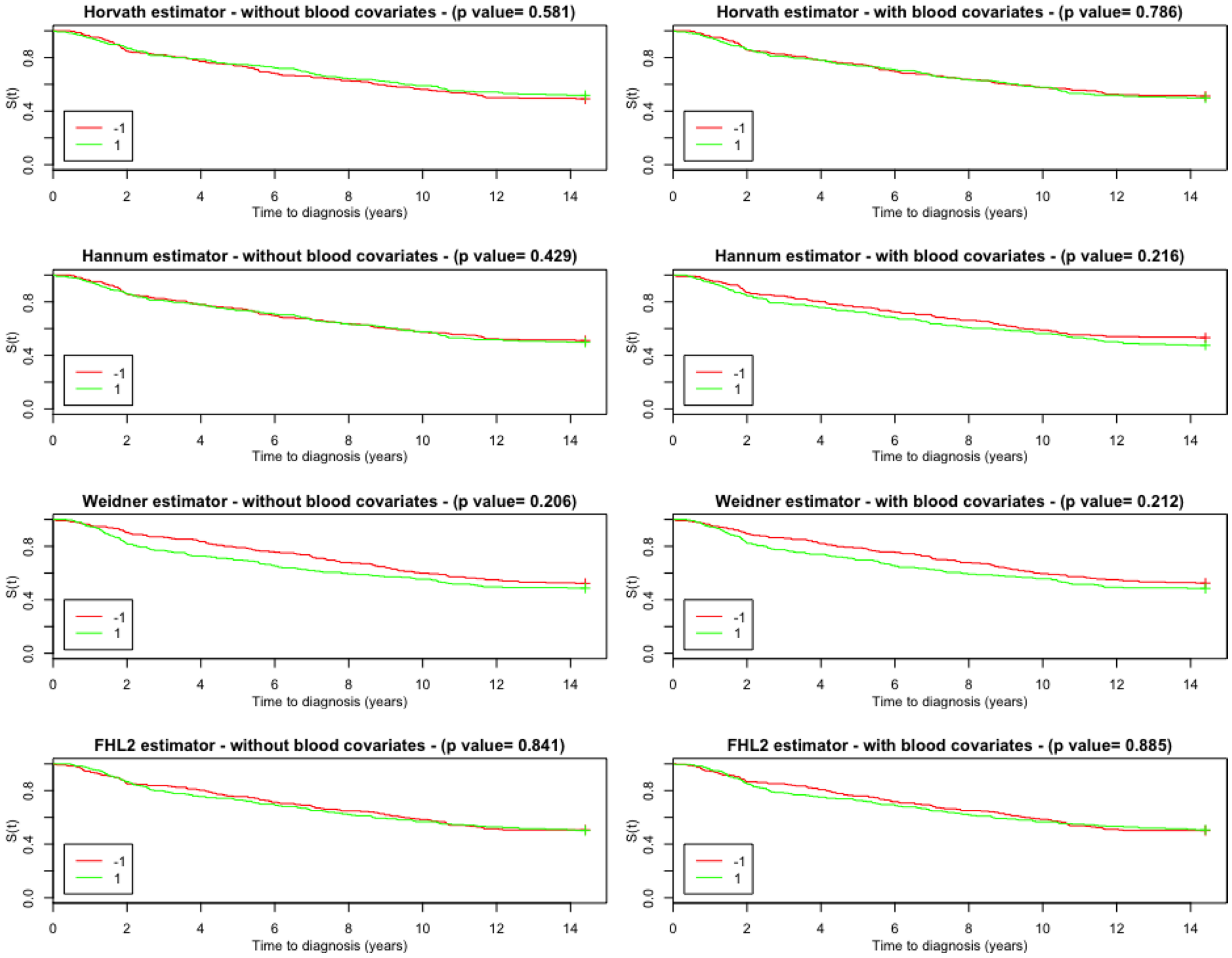

Supplementary Figure 1 : Survival functions for subjects belonging to the breast cancer group (including controls) estimated with Kaplan-Meier method. Results are shown separately for accelerated (1) and decelerated (–1) age subjects, with age acceleration computed considering Horvath, Hannum, Weidner and FHL2 estimators that showed non-significant differences between cases and controls. In each chart title, we reported the Log-Rank test *p*-values comparing survival curves.

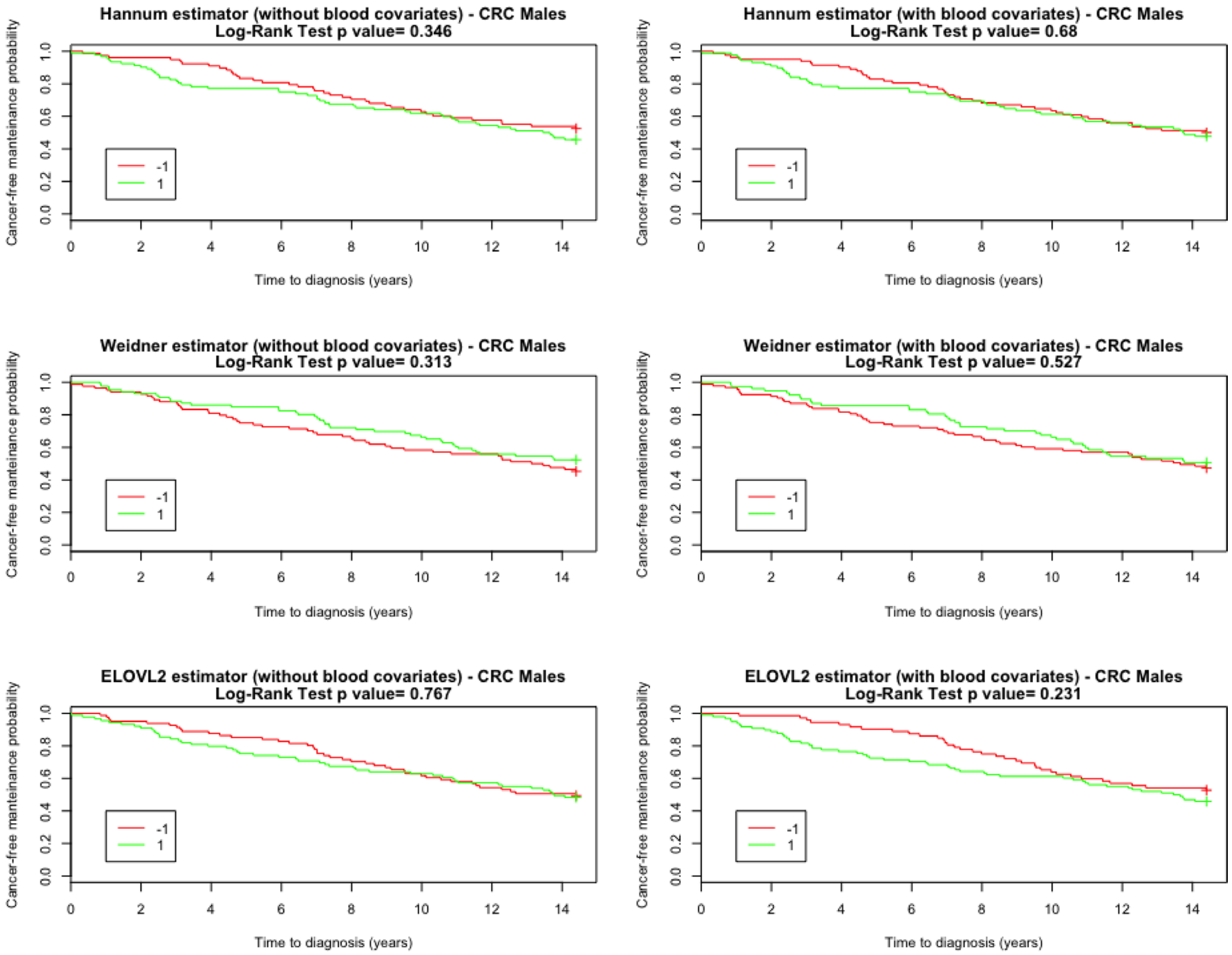

Supplementary Figure 2: Survival functions for subjects belonging to the CRC male cancer group (including controls) estimated with Kaplan-Meier method. Results are shown separately for accelerated (1) and decelerated (–1) age subjects, with age acceleration computed considering the Hannum, Weidner and ELOVL2 estimators that showed non-significant differences between cases and controls. In each chart title, we reported the Log-Rank test *p*-values comparing survival curves.

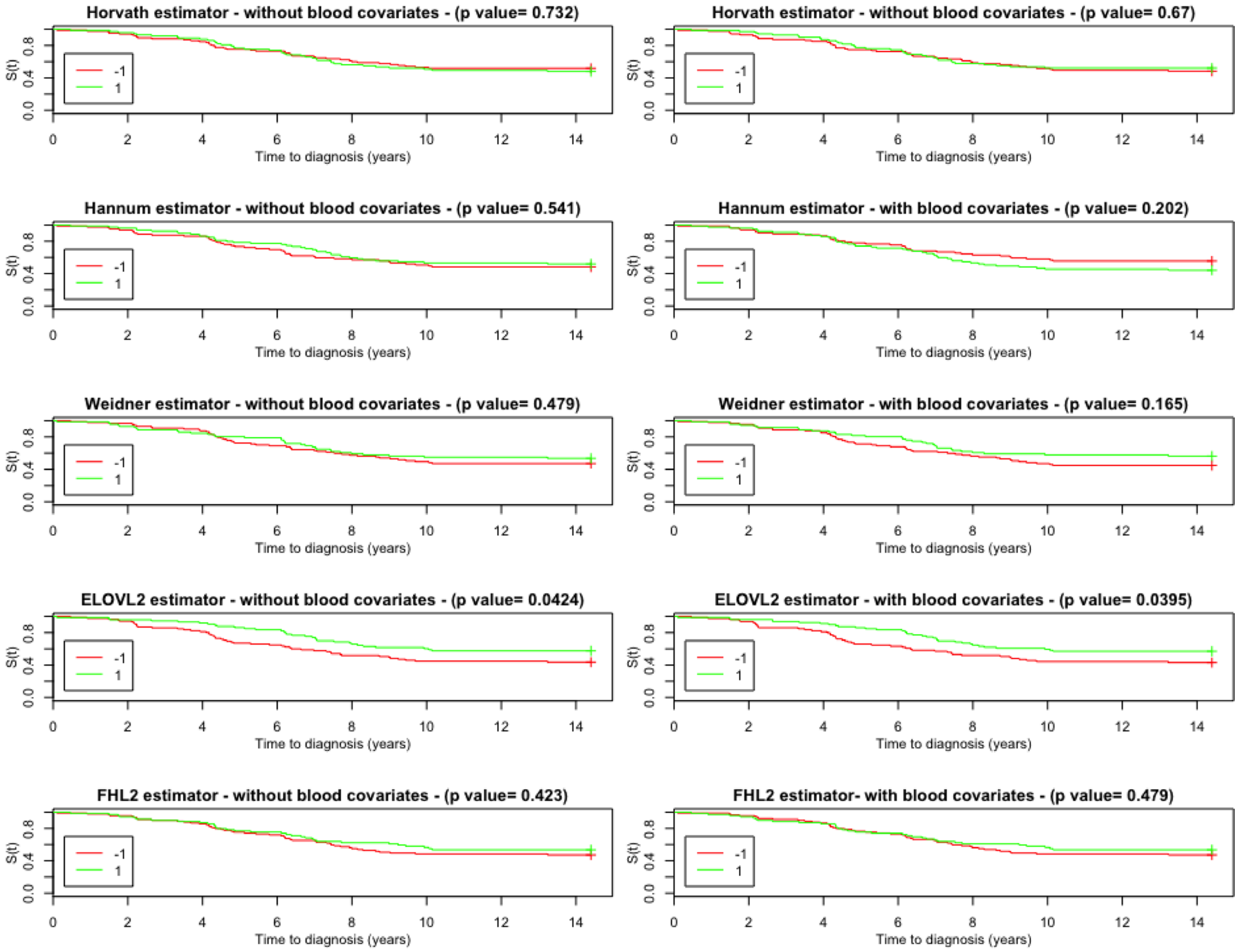

Supplementary Figure 3: Survival functions for subjects belonging to the CRC female cancer group (including controls) estimated with Kaplan-Meier method. Results are shown separately for accelerated (1) and decelerated (–1) age subjects, with age acceleration computed considering all estimators, which showed non-significant differences between cases and controls. In each chart title, we reported the Log-Rank test *p*-values comparing survival curves.
